# Xylazine in Overdose Deaths and Forensic Drug Reports in US States, 2019-2022

**DOI:** 10.1001/jamanetworkopen.2023.50630

**Published:** 2024-01-05

**Authors:** Manuel Cano, Raminta Daniulaityte, Flavio Marsiglia

**Affiliations:** 1 School of Social Work, Arizona State University, Phoenix; 2 College of Health Solutions, Arizona State University, Phoenix; 3 Global Center for Applied Health Research, Arizona State University, Phoenix

## Abstract

**Importance:**

Xylazine is increasingly reported in street drugs and fatal overdoses in the US, yet state-level data are limited, hampering local public health responses.

**Objective:**

To gather available state-level data on xylazine involvement in overdose deaths and in forensic drug reports.

**Design, Setting, and Participants:**

This cross-sectional study was a secondary analysis of 2019 to 2022 data from the National Forensic Laboratory Information System (NFLIS), National Center for Health Statistics, and individual states' medical examiner or public health agency reports. Data were analyzed from August to October 2023.

**Exposure:**

State.

**Main Outcomes and Measures:**

Yearly xylazine-related overdose deaths per 100 000 residents; xylazine NFLIS drug reports, both per 100 000 residents and as a percentage of all NFLIS drug reports (from samples of drugs seized by law enforcement and analyzed by NFLIS-participating laboratories).

**Results:**

A total of 63 state-years were included in analyses of mortality rates, while 204 state-years were included in analyses of NFLIS reports. According to the publicly available data compiled in this study, at least 43 states reported at least 1 xylazine-related overdose death from 2019 to 2022, yet yearly totals of xylazine-related deaths were available for only 21 states. Of states with data available, xylazine-involved overdose death rates were highest in Vermont (10.5 per 100 000 residents) and Connecticut (9.8 per 100 000 residents) in 2022. In 2019, 16 states had zero xylazine reports included in NFLIS reports; in 2022, only 2 states had zero xylazine reports and all but 3 states had recorded an increase in xylazine’s representation in NFLIS reports. In 2022, xylazine represented 16.17% of all NFLIS reports in Delaware and between 5.95% and 7.00% of NFLIS reports in Connecticut, Maryland, District of Columbia, New Jersey, and Rhode Island, yet less than 1.0% of NFLIS reports in 35 different states.

**Conclusions and Relevance:**

In this cross-sectional study of publicly available data on fatal overdoses and drugs analyzed by forensic laboratories, xylazine’s reported presence in overdose deaths and forensic reports was concentrated in the eastern US yet extended across the country to encompass nearly all states. In spite of xylazine’s geographic reach, yearly state-level numbers of xylazine-related overdose deaths were publicly available for less than half of all states.

## Key Points

**Question:**

To what extent is xylazine reported in fatal overdoses and law enforcement drug submissions in different states?

**Findings:**

In this cross-sectional study of US overdose deaths and forensic reports, 16 states had no xylazine forensic reports in 2019, and only 2 states had no xylazine reports in 2022. By 2022 xylazine’s representation in forensic reports had increased in all but 3 states, and at least 43 states reported xylazine-related deaths; among states with data available, xylazine-involved death rates were highest in Vermont and Connecticut in 2022.

**Meaning:**

These results suggest that xylazine forensic reports and overdose deaths are concentrated in eastern states but have increased across the US.

## Teaser

This cross-sectional study of drug overdose deaths in the US examines trends in xylazine-related overdoses and the prevalence of including xylazine in state-level forensic reports.

## Introduction

Xylazine (a sedative and analgesic approved only for veterinary use) has been designated by the US federal government as an emerging threat due to its increasing presence in fatal overdoses.^1^ Xylazine-involved overdose deaths in the US rose from 102 in 2018 to 3468 in 2021,^2^ and 99.1% of these deaths also involved fentanyl.^2^ Xylazine-involved overdose deaths are primarily concentrated in the eastern US,^2^ especially in the Northeast.^3^ Nonetheless, it is less clear to what extent xylazine is involved in overdose deaths within individual states across the US, as xylazine is not uniformly included in every jurisdiction’s postmortem toxicology testing, and counts of xylazine-involved overdose deaths are not accessible via the Centers for Disease Control and Prevention (CDC)’s national mortality database. State-level data on xylazine-involved overdose deaths are currently limited in peer-reviewed publications, including analyses from individual states (Connecticut^4^; West Virginia^5^; Tennessee^6^) and a CDC report^7^ providing ranges (eg, 100 to 499 deaths) of fentanyl-xylazine–positive deaths for 31 states. In a 2022 publication,^8^ data on xylazine-related overdose deaths were available for only 14 jurisdictions in the US (including cities, counties, and states).

In light of gaps in overdose death data, various studies have used law enforcement drug seizure data to assess the presence of different substances in illicit drug supplies^9,10,11,12,13,14,15,16^ and predict overdose mortality rates.^10,12,13,14,15,16,17^ Even though data from forensic reports do not represent a random sample of the illicit drug supply, or even a random sample of all drugs seized,^18^ state- and county-level studies have consistently documented positive associations between overdose mortality rates and numbers or rates of forensic reports of fentanyl and fentanyl-related compounds.^10,12,13,14,16,17,19^ Relatively less has been documented about xylazine forensic reports,^20^ especially at the state level,^19^ yet these data may provide information about the geography of xylazine-adulterated drugs.

Information about xylazine’s presence in street drug supplies and in overdose deaths within individual states is necessary to inform local public health agencies’ and clinicians’ implementation of xylazine-specific harm reduction strategies. Therefore, this cross-sectional, descriptive study compiles and depicts available data on xylazine’s reported involvement in US overdose deaths and forensic drug reports across states and years (2019 to 2022).

## Methods

This study followed Strengthening the Reporting of Observational Studies in Epidemiology (STROBE) reporting guidelines for cross-sectional studies, and no institutional review board review or informed consent was required for this analysis of aggregated, deidentified publicly available mortality data.

### Data Sources

#### Xylazine-Related Overdose Death Data

Because numbers of xylazine-involved overdose deaths are not currently directly identifiable via *International Statistical Classification of Diseases and Related Health Problems, Tenth Revision (ICD-10)* codes^2^ from the CDC’s national mortality database,^21^ we conducted a systematic online search for state-level data on xylazine-related overdose deaths for any year between 2019 and 2022 (following the example of a prior study^8^) (eTables 1-3 in Supplement 1). Limiting our data sources^5,7,8,22,23,24,25,26,27,28,29,30,31,32,33,34,35,36,37,38,39,40,41,42,43,44,45,46,47,48,49^ to official government websites, webpages of entities contracted to analyze statewide overdose data, and peer-reviewed journal articles, we recorded the following: any available state-level yearly count of reported xylazine-related overdose deaths; any other report of xylazine-related deaths (for states where yearly counts were not provided); and information regarding whether the reported deaths were xylazine-involved (ie, xylazine was identified as a contributing cause of death) or xylazine-positive (ie, all overdose deaths where xylazine was detected, whether or not xylazine was identified as a contributing cause of death^7^).

#### Xylazine Forensic Drug Report Data

Forensic drug report data were obtained from the National Forensic Laboratory Information System (NFLIS) Public Data Query System,^50^ which provides public access data from law enforcement drug seizure samples submitted to and analyzed by participating US forensic laboratories.^51^ Results from approximately 95% of analyzed drug submissions are available in NFLIS within 3 months after the end of each year, and the remaining data are updated as available.^51^ The data used in this study comprised yearly counts of total drug reports, as well as xylazine reports specifically, recorded in the NFLIS-Drug Public Data Query System by October 23, 2023, for each of the 50 states and the District of Columbia (DC) from 2019 to 2022. Each drug report represents 1 drug identified during analyses of drug samples seized by law enforcement.^51^ NFLIS-participating laboratories account for more than 98% of the national drug caseload,^51^ yet unweighted drug report counts are not considered nationally representative of all forensic reports.^18,51^ The NFLIS drug reports available from the Public Data Query System (used in the present study) are unweighted, raw counts distinct from the statistically adjusted estimates reported in Drug Enforcement Administration publications.^51^ Nonetheless, multiple studies indicate that these unweighted counts or rates of NFLIS drug reports are associated with state-year overdose mortality rates.^10,14,16,19^

### Statistical Analysis

For each state and year with data available, we calculated xylazine-related overdose mortality rates per 100 000 population, using reported numbers of xylazine-related overdose deaths retrieved during our systematic online search and population estimates from the National Center for Health Statistics.^21^ Because the xylazine-related overdose death rates were based on data reported by individual states that use varying toxicology and reporting procedures, we did not conduct formal analyses of differences between states or over time, instead providing estimated rates along with information regarding whether the state reported all xylazine-positive deaths or only xylazine-involved deaths (based on CDC definitions of xylazine-positive and xylazine-involved).^7^

Next, we calculated xylazine NFLIS drug report rates (per 100 000 population) for each state and year, also using population estimates from the National Center for Health Statistics,^21^ depicting rates in choropleth maps. Because NFLIS drug reports represent raw counts that do not account for laboratory nonparticipation or variation in states’ levels of drug trafficking or law enforcement activities,^18^ we also examined an additional measure that is potentially less sensitive to these sources of variation: xylazine NFLIS drug reports as a percentage of all NFLIS drug reports, calculated for each state *i* and year *j* as follows:



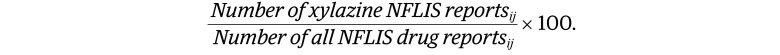



Prior studies have found that both drug report counts (or rates) and percentages are associated with state-level overdose mortality,^10,12,14,19^ yet counts and rates are presumably influenced by states' levels of drug enforcement and drug commerce, while percentage-based measures may theoretically reflect the composition of illicit drug supplies irrespective of overall levels of law enforcement or drug trafficking. Therefore, in addition to examining xylazine NFLIS report rates, we calculated and plotted xylazine NFLIS reports as a percentage of all NFLIS drug reports for each state and year, additionally calculating absolute change (2022 percentage—2019 percentage) and relative change, calculated with the following formula:



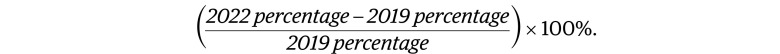



Analyses were completed with RStudio version 1.3.1093 (R Project for Statistical Computing). Data were analyzed from August to October 2023.

## Results

### State-Level Xylazine-Related Overdose Death Rates

We were not able to locate an official record of xylazine-related overdose deaths for 8 states (Alaska, Hawaii, Idaho, Montana, North Carolina, South Dakota, Utah, and Wyoming), yet all other 43 states (including DC) reported at least 1 xylazine-related overdose death between 2019 and 2022 (Table). For many states, the only data that we located comprised ranges of xylazine-involved overdose deaths (eg, 1 to 9, 10 to 99) over 18-month time periods or data from limited samples of deaths analyzed (eg, California, Massachusetts) or specific substate areas (eg, Milwaukee, Wisconsin). In contrast, yearly numbers of reported xylazine-related overdose deaths were located for 21 states, representing 63 state-year observations: 11 states with 2019 data; 15 states with 2020 data; 19 states with 2021 data; and 18 states with 2022 data. Data for most of these states represented numbers of xylazine-involved deaths (where xylazine was not only detected but also identified as a contributing cause of death), while data for fewer states represented xylazine-positive deaths (Maryland, Michigan, Oregon, West Virginia) or did not specify whether xylazine was merely detected or also identified as a contributing cause of death (Alabama, New Jersey). Of the states with yearly numbers available, the highest estimated rates (per 100 000 residents) in 2022 were observed in Vermont (10.5) and Connecticut (9.8), with both states reporting xylazine-involved deaths.

**Table.** Reported Xylazine-Related Overdose Deaths, 2019-2022

State	No. (rate per 100 000 residents)^a^				Additional information	Source
	2019	2020	2021	2022		
AK	NR	NR	NR	NR	NA	NA
AL	NR	NR	14 (0.3)	55 (1.1)	NA	Gulf Coast HIDTA,^30^ 2023
AR	NR	NR	NR	NR	Jan 2021-June 2022: between 1-9 IMF deaths xylazine-positive	Kariisa et al,^7^ 2023
AZ	NR	NR	NR	NR	Jan 2021-June 2022: between 10-99 IMF deaths xylazine-positive	Kariisa et al,^7^ 2023
CA	NR	NR	NR	NR	2021: 0.5% of Sampled deaths xylazine-positive, 0.3% xylazine-involved	CA Department of Public Health,^23^ 2023
CO	NR	NR	NR	NR	Jan 2021-June 2022: between 1-9 IMF deaths xylazine-positive	Kariisa et al,^7^ 2023
CT	71 (2.0)	141 (4.0)	298 (8.3)	354 (9.8)	Xylazine-involved deaths	CT Department of Public Health,^24,25^ 2023
DC	NR	3 (0.4)	6 (0.9)	11 (1.6)	Xylazine-involved deaths	DC Office of the Chief Medical Examiner,^26^ 2023
DE	NR	NR	NR	NR	Jan 2021-June 2022: between 1-9 IMF deaths xylazine-positive	Kariisa et al,^7^ 2023
FL	NR	NR	NR	(1.1)^b^^,^^c^	Xylazine-involved; based on first half of year	FL Department of Law Enforcement,^28^ 2023
GA	NR	15 (0.1)	116 (1.1)	222 (2.1)	Xylazine-involved	GA Department of Public Health,^29^ 2023
HI	NR	NR	NR	NR	NA	NA
IA	NR	NR	NR	NR	Jan 2021-June 2022: between 1-9 IMF deaths xylazine-positive	Kariisa et al,^7^ 2023
ID	NR	NR	NR	NR	NA	NA
IL	53 (0.4)	64 (0.5)	188 (1.5)	NA (2.3)^b^^,^^c^	Xylazine-involved; 2022 rate based on first half of year	Feinberg School of Medicine,^27^ 2023
IN	NR	NR	NR	NR	Jan 2021-June 2022: 82 IMF deaths xylazine-involved	Kariisa et al,^7^ 2023
KS	NR	NR	NR	NR	Jan 2021-June 2022: between 1-9 IMF deaths xylazine-positive	Kariisa et al,^7^ 2023
KY	NR	NR	NR	NR	In 2022: 1-5 xylazine-positive deaths	KY Office of Drug Control Policy,^31^ 2023
LA	3 (0.1)	21 (0.5)	39 (0.8)	25 (0.5)^d^	Xylazine-involved	LA State Board of Medical Examiners,^32^ 2023
MA	NR	NR	NR	NR	In 2022: of opioid deaths with toxicology, 5% xylazine-positive	MA Department of Public Health,^33^ 2023
MD	103 (1.7)	344 (5.7)	495 (8.0)	NR	Xylazine-positive	Friedman et al,^8^ 2022
ME	NR	NR	53 (3.9)	46 (3.4)	Xylazine-involved	Sorg et al,^45^ 2022; Sorg et al,^46^ 2023
MI	NR	NR	60 (0.6)	92 (0.9)	Xylazine-positive	MI Department of Health and Human Services,^34^ 2023
MN	4 (0.1)	8 (0.1)	24 (0.4)	34 (0.6)^d^	Xylazine-involved	MN Department of Health,^35^ 2023
MO	4 (0.1)	2 (0.0)	39 (0.6)	109 (1.8)^d^	Xylazine-involved	Nickelson,^40^ 2023
MS	NR	NR	NR	NR	Jan 2020-Jun 2022: 19 Xylazine-involved deaths	MS State Department of Health,^36^ 2023
MT	NR	NR	NR	NR	NA	NA
NC	NR	NR	NR	NR	NA	NA
ND	NR	NR	NR	NR	Jan 2019-July 2023: 9 Xylazine-positive deaths	ND Department of Health and Human Services,^41^ 2023
NE	NR	NR	NR	NR	2021-2022: 4 Xylazine-involved deaths	NE Department of Health and Human Services,^37^ 2023
NH	NR	NR	NR	3 (0.2)^d^	Xylazine-involved	NH Office of Chief Medical Examiner,^38^ 2023
NJ	13 (0.1)	34 (0.4)	226 (2.4)^d^	210 (2.3)^d^	NA	Bureau of Justice Assistance,^22^ 2023
NM	NR	NR	NR	NR	Jan 2021-June 2022: between 1-9 IMF deaths xylazine-positive	Kariisa et al,^7^ 2023
NV	NR	NR	NR	NR	Jan 2021-June 2022: between 1-9 IMF deaths xylazine-positive	Kariisa et al,^7^ 2023
NY	NR	NR	NR	NR	2021: 429 xylazine-involved opioid deaths in NYC; 135 in New York State outside NYC	NY State Department of Health,^39^ 2023
OH	15 (0.1)	45 (0.4)	75 (0.6)	119 (1.0)^d^	Xylazine-involved	OH Department of Health,^42^ 2023
OK	NR	NR	NR	NR	Jan 2021-June 2022: between 1-9 IMF deaths xylazine-positive	Kariisa et al,^7^ 2023
OR	NR	2 (0.0)	9 (0.2)	9 (0.2)^d^	Xylazine-positive	OR Health Authority,^43^ 2023
PA	259 (2.0)	377 (2.9)	576 (4.4)	760 (5.9)	Xylazine-involved	PA Department of Health,^44^ 2023
RI	NR	NR	NR	NR	Jan 2021-June 2022: between 10-99 IMF deaths xylazine-positive	Kariisa et al,^7^ 2023
SC	NR	NR	NR	NR	Jan 2021-June 2022: 178 IMF deaths involving xylazine	Kariisa et al,^7^ 2023
SD	NR	NR	NR	NR	NA	NA
TN	NR	56 (0.8)	94 (1.3)	NR	Xylazine-involved	TN Department of Health,^47^ 2022
TX	NR	NR	11 (0.0)	19 (0.1)	Xylazine-involved	TX Department of State Health Services,^48^ 2023
UT	NR	NR	NR	NR	NA	NA
VA	NR	NR	NR	NR	Jan 2021-June 2022: between 10-99 IMF deaths xylazine-positive	Kariisa et al,^7^ 2023
VT	6 (1.0)	5 (0.8)	29 (4.5)	68 (10.5)	Xylazine-involved	VT Department of Health,^49^ 2023
WA	NR	NR	NR	NR	Jan 2021-June 2022: between 1-9 IMF deaths xylazine-positive	Kariisa et al,^7^ 2023
WI	NR	NR	NR	NR	2019-2020: 6 xylazine-positive deaths reported in Milwaukee	Friedman et al,^8^ 2022
WV	10 (0.6)	67 (3.8)	(4.5)^b^^,^^c^	NR	Xylazine-positive	Sibbesen et al,^5^ 2023
WY	NR	NR	NR	NR	NA	NA

Abbreviations: IMF, illicitly manufactured fentanyl; NA, not available; NR, not reported.

^a^ Death counts were most recently accessed from each source October 27 to 30, 2023. Rates are per 100 000 population and calculated using population estimates from the National Center for Health Statistics.

^b^ Totals provided for a half-year period, and not included.

^c^ Data provided for half of year only.

^d^ Data source indicates this figure is based on provisional data.

### State-Level Xylazine NFLIS Drug Report Rates

**Figure 1.**
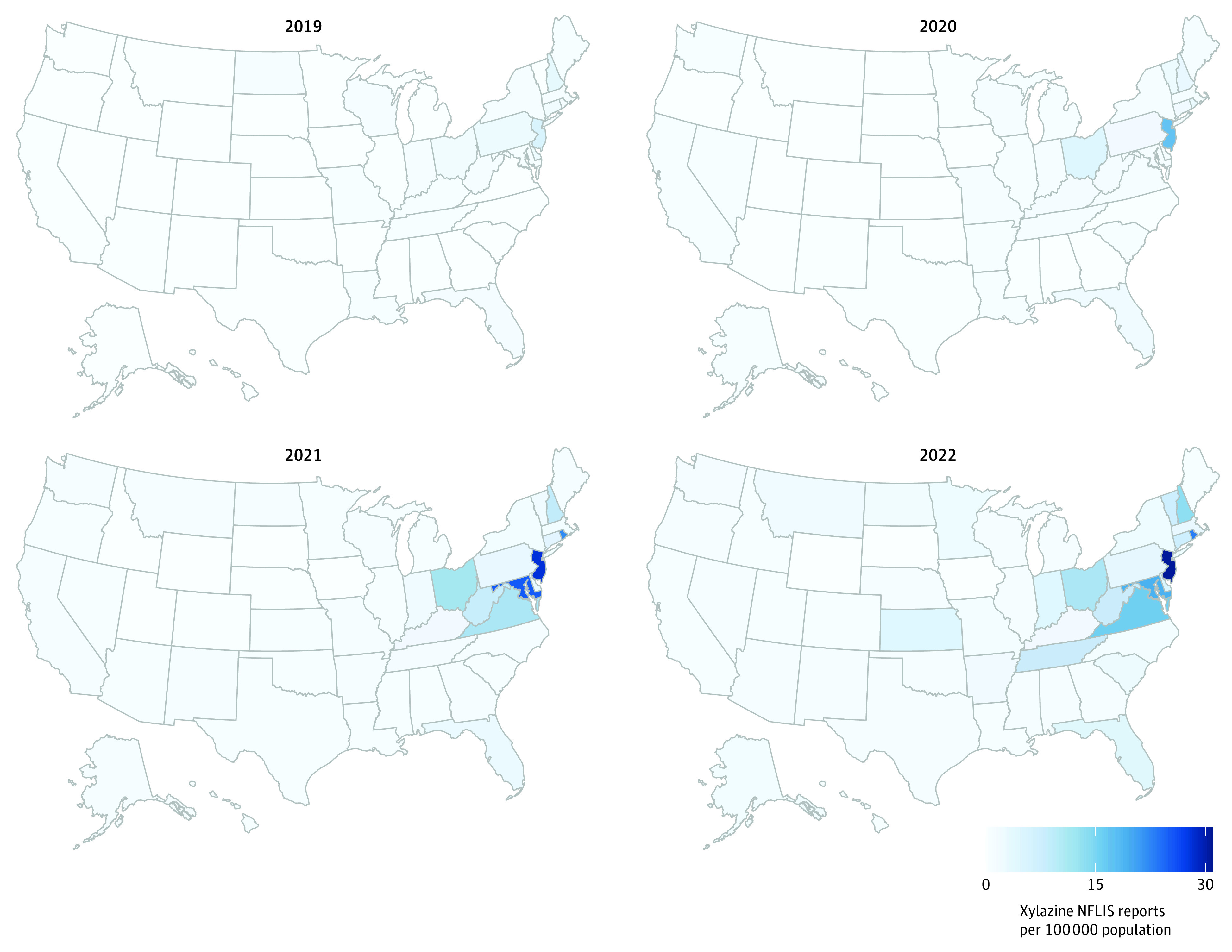
Xylazine NFLIS Drug Report Rates (per 100 000 Population), US, 2019-2022 Source: National Forensic Laboratory Information System (NFLIS)-Drug Public Data Query System as accessed on October 23, 2023, for each of the 50 states and District of Columbia; population estimates (to compute rates) from the National Center for Health Statistics.

In 2019, relatively low rates of xylazine NFLIS reports were observed; all states had a rate below 6 xylazine NFLIS reports per 100 000 residents, and 16 states had zero NFLIS xylazine reports (Figure 1; eTable 4 in Supplement 1). In contrast, in 2022, only 2 states had zero xylazine NFLIS drug reports, and the highest rates of xylazine NFLIS reports (per 100 000 residents) were observed in New Jersey (30.52), Rhode Island (22.82), Maryland (18.91), Virginia (15.47), New Hampshire (13.10), and Ohio (10.87). In 2019, xylazine NFLIS reports were limited to a few northeastern states; by 2022, xylazine NFLIS reports were still concentrated in northeastern states yet had extended to states south and west.

**Figure 2.**
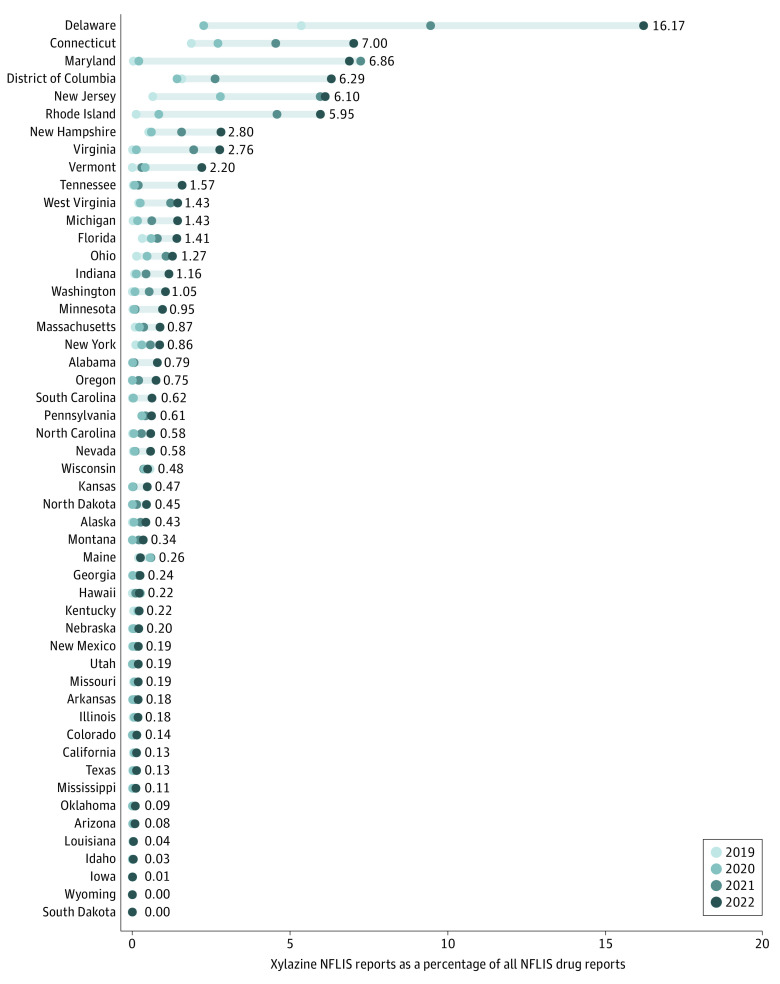
Xylazine NFLIS Drug Reports as a Percentage of all NFLIS Drug Reports, US, 2019-2022 Source: National Forensic Laboratory Information System (NFLIS)-Drug Public Data Query System as accessed on October 23, 2023, for each of the 50 states and District of Columbia.

In 2022, xylazine represented 16.17% of all NFLIS reports in Delaware and between 5.95% and 7.00% of NFLIS reports in Connecticut, Maryland, DC, New Jersey, and Rhode Island, yet less than 1.00% of NFLIS reports in 35 different states (Figure 2; eTable 5 in Supplement 1). Delaware, Maryland, Rhode Island, New Jersey, Connecticut, DC, and Virginia had the highest absolute change in xylazine reports as a percentage of all NFLIS drug reports (2019-2022), while Virginia, Maryland, North Carolina, Michigan, Kansas, Alabama, Minnesota, and Tennessee had the highest relative change. Only 1 state, Wisconsin, recorded a decrease in xylazine’s representation in NFLIS drug reports from 2019 (0.56% of drug reports) to 2022 (0.48% of drug reports), and 2 states reported no increase (0% in 2019 to 0% in 2022; South Dakota and Wyoming) yet in 30 states, across all regions, xylazine reports (as a percentage of all NFLIS drug reports) more than doubled.

## Discussion

Results of this descriptive, cross-sectional study highlighted increasing reports of xylazine in overdose deaths and forensic laboratory data in the US. Although it is unclear to what extent these increases may reflect the expansion of testing for xylazine,^2^ regional studies have documented increases in xylazine detection in overdose deaths even in jurisdictions where testing for xylazine has been consistently implemented across multiple years.^4,52^ This study’s results add to existing research in several ways. First, although xylazine is geographically concentrated in drug supplies in the eastern US, xylazine detection in NFLIS drug reports has risen in states across the US; from 2019 to 2022, the percentage of NFLIS drug reports positive for xylazine increased in all but 3 states and more than doubled in 30 different states spanning all regions. Second, 43 states reported at least 1 xylazine-related death, yet yearly numbers of xylazine-related overdose deaths were publicly available for less than half of all states, and xylazine-related overdose deaths were inconsistently reported between and within states. Overall, study findings underscore the need for more state-level (and community-level) data on xylazine-related overdoses to inform local overdose response initiatives.

Expanding and standardizing postmortem testing and reporting procedures across US jurisdictions has the potential to support more uniformly available and complete data on xylazine-involved deaths.^53,54^ In this study’s compilation of publicly available data from government agencies and peer-reviewed journal articles, we were able to locate an official record of at least 1 xylazine-related overdose death in 84% of states, yet in many of these states, the only publicly available data comprised estimated ranges of deaths, data from limited samples of deaths analyzed, or data confined to substate areas such as individual cities or counties. Yearly state-level numbers of reported xylazine-related overdose deaths were available only for 21 states, potentially reflecting limited testing for xylazine,^2^ as well as xylazine’s status as a relatively newer adulterant. Researchers have documented substantial differences between and within states in terms of death investigation systems, toxicology procedures, and the completeness of drug reporting on death certificates, frequently in the context of the underreporting of opioid-involved overdose deaths.^55,56^ Our study’s results suggest that in addition to these well-documented differences, states also differ in terms of what types of xylazine-related deaths are reported publicly, with some state-level data representing all overdose deaths in which xylazine was detected (xylazine-positive deaths), and other data identifying only deaths in which xylazine was designated as a contributing cause of death (xylazine-involved deaths). A 2023 report from the CDC^7^ documented differences between several states in the extent to which xylazine was recorded on the death certificates of xylazine-positive deaths, suggesting the utility of reporting comprehensive toxicology data instead of relying only on the drugs listed on death certificates for surveillance.

Data from law enforcement drug reports, as well as community drug-checking programs^57,58,59^ and health care settings,^60,61^ may help supplement the limited xylazine mortality data available to gauge local levels of xylazine risk. In our analysis of NFLIS drug reports, xylazine reports were limited to a few northeastern states in 2019, yet by 2022, xylazine drug reports had extended to states south and west but were still concentrated in northeastern states. Maryland, Connecticut, and Pennsylvania have been identified as states with the highest xylazine positivity in fentanyl deaths,^7^ and Pennsylvania, North Carolina, Ohio, and Maryland were recently identified as states with the highest xylazine positivity in fentanyl-positive urine drug tests.^62^ Adding to this list of states identified as hotspots for xylazine risk, this study’s results indicate that Vermont has recorded some of the highest recent rates of xylazine-involved overdose deaths, and Delaware, DC, New Jersey, and Rhode Island accompany Connecticut and Maryland as states with the highest percentage of xylazine in NFLIS drug reports. States with high percentages of xylazine in forensic drug reports may represent areas where xylazine overdose surveillance is particularly relevant, yet in our study, several states with relatively high percentages of xylazine in NFLIS drug reports (eg, Rhode Island, Virginia) had no publicly reported totals of yearly xylazine-related overdose deaths.

Xylazine’s presence in drug supplies represents only 1 potential predictor of deaths involving xylazine, and law enforcement submissions in any given state (especially at points of entry or high trafficking areas) may include drugs originally in route to other states’ street drug supplies. In the present study, for example, New Jersey (a state identified as a major entry point for drugs^63^) represented the state with the highest xylazine NFLIS drug reports per capita, yet New Jersey was not the state with the highest reported xylazine-related overdose death rate. Conversely, Pennsylvania has been identified as the epicenter of xylazine-adulterated drugs, with high xylazine-positivity in fentanyl deaths^7^ and in urine tests,^62^ yet in our study, Pennsylvania was not one of the states with the highest rates or percentages of xylazine NFLIS drug reports.

In communities with xylazine prevalent in drug supplies, overdose prevention efforts may be strengthened via the evaluation, optimization, and incorporation of xylazine-specific harm reduction strategies. Recently developed xylazine test strips represent 1 such potential harm reduction strategy^64^; research in this area is still nascent,^65,66^ especially relative to research on fentanyl test strips.^67^ In addition to test strips, integrating xylazine information in overdose prevention training may better prepare individuals who use drugs and other lay and professional overdose responders.^68^ The opioid reversal medication naloxone can counteract the respiratory depression caused by the opioids that commonly accompany xylazine but cannot address the effects of xylazine itself (because xylazine is not an opioid^53^), so lay responders are often advised to not only administer naloxone but also call emergency medical services and provide rescue breathing,^69^ and medical professionals may need to provide additional interventions specific to xylazine-adulterated drugs.^1,53^ While arguably not directly related to overdose, wound care also represents a harm reduction priority particularly relevant for individuals who use drugs containing xylazine due to the severe skin ulcerations that may accompany use.^68,69,70^ These skin ulcerations, as well as xylazine withdrawal symptoms, also complicate the provision of effective substance use disorder treatment,^1,68^ necessitating additional research and evaluation to optimize treatment care protocols.^54^

### Limitations

Our study had several limitations. Although the NFLIS reports a 98% participation rate from US forensic laboratories, not every drug seized is analyzed,^51^ and because NFLIS does not represent a random sample of the US illicit drug supply,^18^ it is unclear to what extent these reports reflect overall street drug availability. Drug report counts from the publicly available NFLIS also lack information about the weights or dosage equivalents of the drugs seized or the combinations of substances within a single drug seizure,^51^ and different forensic laboratories employ different procedures for testing and reporting.^18^ In consideration of these data limitations, we examined 2 different xylazine forensic report measures in this study (rates per population and percentage of all NFLIS drug reports) in an effort to better approximate the extent of xylazine’s representation in states’ illicit drug supplies. Multiple studies have documented associations between state-year NFLIS counts, rates, and percentages and drug overdose mortality rates,^10,14,16,19^ suggesting the utility of these measures in spite of their limitations.

In this study, the state-level data on xylazine-related overdose deaths were located via an online search, resulting in death counts obtained from individual states that employ different procedures for death investigations, toxicology, death certificate reporting, and data presentation, precluding formal comparisons of xylazine-related overdose death rates between states or years. Differences in rates of reported xylazine-related overdose deaths may reflect regional and time differences in the extent to which local jurisdictions test for xylazine in postmortem toxicology, and xylazine-related overdose deaths are generally underreported due to limited testing in many jurisdictions.^2^ Finally, data were obtained via a systematic search process and were updated as of October 27 through 30, 2023, yet may not encompass all available data.

## Conclusions

Although xylazine is not currently one of the top drugs contributing to overdose deaths in the US overall, xylazine-related overdose deaths are relatively high within certain communities,^7,52^ rapidly increasing, and likely underestimated due to limited testing.^2,53^ Moreover, xylazine’s presence in illicit drug supplies has extended across the country, no longer limited to the Northeast.^20,62,71^ Timely identification of xylazine in local drug supplies and overdoses represents a first step in responding to this emerging threat,^1^ followed by the evaluation, optimization, dissemination, and implementation of xylazine-specific harm reduction strategies, including those pioneered by frontline workers in regions with a longer history and higher prevalence of xylazine in street drug supplies.^68^

## Notes

Supplement 1. **eTable 1.** Search Strategy Used in Online Search for State-Level Xylazine-Related Overdose Death Data **eTable 2.** General Inclusion and Exclusion Criteria for Sources of State-Level Xylazine-Related Overdose Death Data **eTable 3.** Inclusion and Exclusion Criteria for Yearly State-Level Counts of Xylazine-Related Overdose Deaths **eTable 4.** Xylazine NFLIS Drug Report Rates (per 100 000 Residents) by State and Year, 2019-2022 **eTable 5.** Xylazine NFLIS Reports as a Percentage of All NFLIS Drug Reports, by State and Year, With Absolute and Relative Change, 2019-2022

Supplement 2.
Data Sharing Statement
