# Supplementary material for: Xylazine in Overdose Deaths and Forensic Drug Reports in US States, 2019-2022
Source: JAMA Netw Open. 2024 Jan 5;7(1):e2350630. doi: 10.1001/jamanetworkopen.2023.50630 (PMC10770774; doi:10.1001/jamanetworkopen.2023.50630)
Supplement: Supplement 1. — eTable 1. Search Strategy Used in Online Search for State-Level Xylazine-Related Overdose Death Data eTable 2. General Inclusion and Exclusion Criteria for Sources of State-Level Xylazine-Related Overdose Death Data eTable 3. Inclusion and Exclusion Criteria for Yearly State-Level Counts of Xylazine-Related Overdose Deaths eTable 4. Xylazine NFLIS Drug Report Rates (per 100 000 Residents) by State and Year, 2019-2022 eTable 5. Xylazine NFLIS Reports as a Percentage of All NFLIS Drug Reports, by State and Year, With Absolute and Relative Change, 2019-2022 [file jamanetwopen-e2350630-s001.pdf]

Supplemental Online Content

Cano M, Daniulaityte R, Marsiglia F. Xylazine in overdose deaths and forensic drug reports in US states, 2019-2022. *JAMA Netw Open.* 2024;7(1):e2350630. doi:10.1001/jamanetworkopen.2023.50630

eTable 1. Search Strategy Used in Online Search for State-Level Xylazine-Related Overdose Death Data

eTable 2. General Inclusion and Exclusion Criteria for Sources of State-Level Xylazine-Related Overdose Death Data

eTable 3. Inclusion and Exclusion Criteria for Yearly State-Level Counts of Xylazine-Related Overdose Deaths

eTable 4. Xylazine NFLIS Drug Report Rates (per 100 000 Residents) by State and Year, 2019-2022

eTable 5. Xylazine NFLIS Reports as a Percentage of All NFLIS Drug Reports, by State and Year, With Absolute and Relative Change, 2019-2022

This supplemental material has been provided by the authors to give readers additional information about their work.

© 2024 Cano M et al. *JAMA Network Open.*

eTable 1. Search strategy used in online search for state-level xylazine-related overdose death data.

Search Engine/Database	Search Terms
PubMed	xylazine AND deaths AND (drug-related OR overdose)
Google	site:[state name/name abbreviation].gov “xylazine”<br>[“state name”] “xylazine” “deaths”<br>[state name] overdose data xylazine<br>[state name] xylazine<br>[state name] overdose data<br>[state name] State Unintentional Drug Overdose Reporting System<br>[state name] medical examiner report

© 2024 Cano M et al. *JAMA Network Open.*

eTable 2. General inclusion and exclusion criteria for sources of state-level xylazine-related overdose death data.

Eligible Sources	✓	Government website
	✓	Agency/educational institution contracted to provide data analysis for state health department
	✓	Peer-reviewed journal article
	✓	Report from the National Center for Health Statistics/Centers for Disease Control and Prevention
Ineligible Sources	×	Any other source (e.g., news report, private agency/service provider)

© 2024 Cano M et al. *JAMA Network Open.*

eTable 3. Inclusion and exclusion criteria for yearly state-level counts of xylazine-related overdose deaths

Included	✓	Counts for one-year time periods or half of a year[a]
	✓	Counts for xylazine-positive or xylazine-involved deaths
	✓	Counts for xylazine overdose deaths, xylazine-opioid deaths, or xylazine-fentanyl deaths[b]
	✓	Finalized data or “preliminary” or “provisional” data[c]
	✓	Deaths of unintentional/undetermined intent or all intents[d]
Excluded[e]	×	Data from multiple years combined, or any time range other than one year or one half of a year
	×	Data without a clear time range specified
	×	Ranges of numbers (e.g., 1-19)
	×	Counts or percentages from partial samples of overdose deaths or substate areas

Notes. [a]When provided, a count for a half year was used to calculate a rate for half of the year, identified as a half-year in Table 1 in the main document. [b]Deaths involving xylazine and opioids, or xylazine and fentanyl, were considered comparable to deaths involving xylazine in general, based on data from the National Center for Health Statistics indicating that more than 99% of xylazine-involved overdose deaths also involve fentanyl (an opioid). [c]Preliminary and provisional data were included for past years, since the National Center for Health Statistics also reports provisional mortality data, and in many cases, the data are nearly complete. [d]Deaths of any intent (e.g., unintentional/undetermined only, or all intents) were included since data from the National Center for Health Statistics indicates that approximately 91-97% of all drug overdose deaths are unintentional/undetermined intent. [e]These types of data were excluded from yearly state-level rate calculations but were provided in Table 1 under the “additional information” column as supplementary data.

eTable 4. Xylazine NFLIS drug report rates (per 100,000 residents) by state and year, 2019-2022.

State	2019	2020	2021	2022
AK	0.00	0.14	0.82	1.23
AL	0.10	0.04	0.30	0.77
AR	0.07	0.16	0.73	1.62
AZ	0.10	0.04	0.23	0.37
CA	0.10	0.22	0.38	0.30
CO	0.00	0.00	0.09	0.28
CT	1.54	1.60	3.77	6.96
DC	2.69	1.82	2.54	6.72
DE	1.85	0.61	2.49	4.88
FL	1.13	1.73	2.50	3.94
GA	0.02	0.04	0.66	0.50
HI	0.00	0.07	0.14	0.21
IA	0.03	0.00	0.00	0.03
ID	0.00	0.00	0.05	0.21
IL	0.09	0.19	0.21	0.51
IN	0.46	0.46	1.76	4.19
KS	0.07	0.03	0.20	4.06
KY	0.29	1.09	1.37	1.37
LA	0.22	0.24	0.22	0.28
MA	0.45	0.71	1.26	2.68
MD	0.20	0.81	25.14	18.91
ME	0.22	0.74	0.80	0.15
MI	0.04	0.20	0.25	0.51
MN	0.04	0.11	0.21	1.94
MO	0.24	0.41	0.49	1.04
MS	0.00	0.10	0.24	0.41
MT	0.00	0.00	1.00	1.72
NC	0.01	0.10	0.58	1.04
ND	0.39	0.00	0.65	1.55
NE	0.00	0.05	0.10	0.51
NH	2.72	2.71	8.35	13.10
NJ	5.19	16.75	28.00	30.52
NM	0.00	0.09	0.57	0.95
NV	0.06	0.13	0.19	0.57
NY	0.36	0.62	1.28	1.65
OH	1.48	4.51	11.17	10.87
OK	0.00	0.03	0.13	0.65
OR	0.00	0.00	0.31	0.78
PA	2.24	1.36	2.63	3.17
RI	0.47	2.55	21.81	22.82
SC	0.00	0.17	0.17	2.18
SD	0.00	0.00	0.00	0.00
TN	0.18	0.36	1.10	7.44

© 2024 Cano M et al. *JAMA Network Open.*

TX	0.03	0.11	0.17	0.42
UT	0.00	0.00	0.03	0.18
VA	0.06	0.77	10.90	15.47
VT	0.00	2.25	1.70	6.97
WA	0.00	0.17	0.54	1.18
WI	0.81	0.50	0.51	0.61
WV	0.95	1.12	7.91	7.80
WY	0.00	0.00	0.00	0.00

Note. Cells shaded in darker colors highlight higher values. Rates based on drug report counts retrieved from the National Forensic Laboratory Information System (NFLIS) on October 23, 2023 and population estimates from the National Center for Health Statistics.

© 2024 Cano M et al. *JAMA Network Open.*

eTable 5. Xylazine NFLIS reports as a percentage of all NFLIS drug reports, by state and year, with absolute and relative change, 2019-2022.

State	2019	2020	2021	2022	Absolute Change, 2019-2022	Relative/Percent Change, 2019-2022
AK	0.000	0.049	0.260	0.427	0.427	*
AL	0.013	0.006	0.054	0.790	0.777	5906.506
AR	0.005	0.013	0.064	0.182	0.177	3272.602
AZ	0.021	0.009	0.058	0.083	0.062	293.950
CA	0.030	0.062	0.121	0.133	0.103	340.299
CO	0.000	0.000	0.034	0.140	0.140	*
CT	1.864	2.707	4.535	7.001	5.137	275.529
DC	1.565	1.413	2.615	6.294	4.729	302.135
DE	5.341	2.256	9.434	16.172	10.830	202.769
FL	0.313	0.595	0.792	1.407	1.093	349.196
GA	0.005	0.012	0.201	0.243	0.238	4387.072
HI	0.000	0.256	0.106	0.222	0.222	*
IA	0.005	0.000	0.000	0.006	0.001	28.858
ID	0.000	0.000	0.007	0.030	0.030	*
IL	0.025	0.064	0.068	0.178	0.153	614.954
IN	0.072	0.126	0.432	1.157	1.085	1506.688
KS	0.008	0.004	0.020	0.471	0.463	5989.740
KY	0.041	0.174	0.196	0.216	0.174	420.906
LA	0.019	0.021	0.021	0.036	0.016	84.905
MA	0.087	0.224	0.358	0.874	0.788	909.955
MD	0.032	0.204	7.223	6.865	6.833	21303.554
ME	0.194	0.556	0.586	0.255	0.061	31.378
MI	0.023	0.164	0.613	1.430	1.407	6145.606
MN	0.016	0.052	0.086	0.954	0.938	5871.567
MO	0.036	0.068	0.072	0.186	0.150	422.448
MS	0.000	0.018	0.040	0.113	0.113	*
MT	0.000	0.000	0.206	0.344	0.344	*
NC	0.006	0.045	0.287	0.581	0.575	9762.730
ND	0.041	0.000	0.135	0.447	0.406	998.436
NE	0.000	0.019	0.040	0.202	0.202	*
NH	0.517	0.601	1.556	2.803	2.286	441.967
NJ	0.648	2.784	5.942	6.100	5.452	841.893
NM	0.000	0.023	0.110	0.189	0.189	*
NV	0.027	0.064	0.085	0.576	0.549	1996.159
NY	0.106	0.296	0.568	0.863	0.757	715.617
OH	0.132	0.465	1.064	1.268	1.136	860.091
OK	0.000	0.005	0.019	0.093	0.093	*
OR	0.000	0.000	0.199	0.749	0.749	*

© 2024 Cano M et al. *JAMA Network Open.*

PA	0.387	0.297	0.416	0.606	0.219	56.441
RI	0.120	0.836	4.574	5.948	5.828	4855.984
SC	0.000	0.034	0.031	0.620	0.620	*
SD	0.000	0.000	0.000	0.000	0.000	*
TN	0.027	0.067	0.188	1.573	1.546	5703.370
TX	0.008	0.029	0.048	0.133	0.125	1601.605
UT	0.000	0.000	0.030	0.188	0.188	*
VA	0.008	0.126	1.939	2.764	2.756	33541.733
VT	0.000	0.407	0.298	2.196	2.196	*
WA	0.000	0.084	0.532	1.047	1.047	*
WI	0.558	0.383	0.358	0.483	-0.075	-13.398
WV	0.198	0.250	1.208	1.434	1.237	625.944
WY	0.000	0.000	0.000	0.000	0.000	*

Note. *Denotes that percent change is undefined due to a starting value (2019 value) of zero. Absolute change denotes annual value in 2022 minus value in 2019, for each state. Percent change denotes annual value in 2022 minus value in 2019, divided by value in 2019, multiplied by 100. Darker blue highlights higher values across states, 2019-2022; darker green highlights higher absolute change values; darker orange indicates higher percent change values. Data based on drug report counts retrieved from the National Forensic Laboratory Information System (NFLIS) on October 23, 2023.

© 2024 Cano M et al. *JAMA Network Open.*
