# Supplementary material for: Xylazine in Overdose Deaths and Forensic Drug Reports in US States, 2019-2022
Source: JAMA Netw Open. 2024 Jan 5;7(1):e2350630. doi: 10.1001/jamanetworkopen.2023.50630 (PMC10770774; doi:10.1001/jamanetworkopen.2023.50630)
Supplement: Supplement 2. — Data Sharing Statement [file jamanetwopen-e2350630-s002.pdf]

Data Sharing Statement

Cano. Xylazine in Overdose Deaths and Forensic Drug Reports in US States, 2019-2022. *JAMA Netw Open*. Published January 05, 2024. doi:10.1001/jamanetworkopen.2023.50630

Data
Data available: No

Additional Information
Explanation for why data not available: All data used in the study are publicly-accessible online from the sources cited in the manuscript.
